# Notch ligand Delta-like 1 as a novel molecular target in childhood neuroblastoma

**DOI:** 10.1186/s12885-017-3340-3

**Published:** 2017-05-19

**Authors:** P. Bettinsoli, G. Ferrari-Toninelli, S. A. Bonini, C. Prandelli, M. Memo

**Affiliations:** Department of Molecular and Translational Medicine, University of Brescia Medical School, Viale Europa, 11 Brescia, Italy

**Keywords:** Neuroblastoma, Notch pathway, Delta-like 1, miRNAs, Molecular target

## Abstract

**Background:**

Neuroblastoma is the most common extracranial solid malignancy in childhood, responsible for 15% of all pediatric cancer deaths. It is an heterogeneous disease that does not always respond to classical therapy; so the identification of new and specific molecular targets to improve existing therapy is needed. We have previously demonstrated the involvement of the Notch pathway in the onset and progression of neuroblastoma. In this study we further investigated the role of Notch signaling and identified Delta-like 1 (*DLL1*) as a novel molecular target in neuroblastoma cells with a high degree of *MYCN* amplification, which is a major oncogenic driver in neuroblastoma. The possibility to act on *DLL1* expression levels by using microRNAs (miRNAs) was assessed.

**Methods:**

DLL1 mRNA and protein expression levels were measured in three different neuroblastoma cell lines using quantitative real-time PCR and Western Blot analysis, respectively. Activation of the Notch pathway as a result of increased levels of DLL1 was analyzed by Immunofluorescence and Western Blot methods. In silico tools revealed the possibility to act on *DLL1* expression levels with miRNAs, in particular with the miRNA-34 family. Neuroblastoma cells were transfected with miRNA-34 family members, and the effect of miRNAs transfection on DLL1 mRNA expression levels, on cell differentiation, proliferation and apoptosis was measured.

**Results:**

In this study, the DLL1 ligand was identified as the Notch pathway component highly expressed in neuroblastoma cells with *MYCN* amplification. In silico analysis demonstrated that *DLL1* is one of the targets of miRNA-34 family members that maps on chromosome regions that are frequently deregulated or deleted in neuroblastoma. We studied the possibility to use miRNAs to target *DLL1*. Among all miRNA-34 family members, miRNA-34b is able to significantly downregulate DLL1 mRNA expression levels, to arrest cell proliferation and to induce neuronal differentiation in malignant neuroblastoma cells.

**Conclusions:**

Targeted therapies have emerged as new strategies for cancer treatment. This study identified the Notch ligand DLL1 as a novel and attractive molecular target in childhood neuroblastoma and its results could help to devise a targeted therapy using miRNAs.

**Electronic supplementary material:**

The online version of this article (doi:10.1186/s12885-017-3340-3) contains supplementary material, which is available to authorized users.

## Background

Neuroblastoma is an embryonic tumor of the sympathetic nervous system which arises during fetal or early postnatal life from sympathetic cells derived from the neural crest [1]. It is the most common solid extracranial malignancy of childhood and is responsible for 15% of all pediatric cancer deaths. Neuroblastoma is an extremely heterogeneous disease; tumors can spontaneously regress or differentiate, even without therapy, or display a very aggressive malignant phenotype that is poorly responsive to current intensive multimodal therapy [2–4]. Despite high-dose chemotherapy, surgery and radiotherapy, in half of cases the tumor has a survival rate of less than 40% [5]. Unsatisfactory response to classical therapies may be attributable to the clinical, biological and histological heterogeneity of “neuroblastomas”. Thus, the identification of novel selective target molecules is needed, to improve existing therapies and to develop new, specific, innovative and less aggressive therapeutic approaches [6, 7]. Our previous study recognized one of the molecular pathways involved in neuroblastoma to be a component of the Notch signaling pathway [8]. The Notch pathway is an highly conserved cell signaling system that regulates cell fate decisions during embryogenesis, modulates the differentiated state of mature cells and is also one of the main factors in the regulation of “cancer stem cells” [9, 10]. Several studies have demonstrated the importance of Notch signaling in the tumor microenvironment and its involvement in many aspects of the disease: the onset of tumor, angiogenesis, the ability to invade tissues and metastasize [11–16]. Depending on organ and tissue type, Notch signaling can function either as a promoter to support tumor development or as a suppressor to inhibit tumor growth. Deregulated expression of Notch proteins, ligands, and targets has been described in a multitude of solid tumors including renal, lung, pancreatic, hepatocellular and gastric carcinoma, melanoma and medulloblastoma [17]. Notch activation is responsible for increased growth and proliferation of neuroblastoma cell lines. On the contrary, Notch pathway downregulation by gamma-secretase inhibitors causes proliferation arrest and cell differentiation [8]. In this study we wanted to investigate in detail the role of Notch signaling in neuroblastoma and we analyzed the role of Notch pathway components, three receptors (Notch1, Notch 2 and Notch 3) and five canonical ligands (Delta-like 1, Delta-like 2, Delta-like 4, Jagged 1 and Jagged 2), in three neuroblastoma cell lines. We identified DLL1 as the Notch pathway component highly expressed in IMR-32 cells, a cell line with a high degree of *MYCN* amplification. About 20% of neuroblastoma cases are characterized by *MYCN* gene amplification, which has been correlated with tumor progression and is routinely used as a clinical biomarker for treatment stratification [18, 19]. The correlation between Delta-like Notch ligand expression and development of other tumors has already been characterized. Overexpression of DLL1 was identified in choriocarcinoma [20] and hepatocellular carcinoma [21], while Delta-like 4 (*DLL4*) expression was correlated to tumor initiation and progression of glioblastoma [22], poor prognosis in pancreatic cancer [23] and colon cancer [24]. We evaluated the possibility to act on the expression of *DLL1* by using miRNAs. During the past decades the involvement of miRNAs in several human diseases, including cancer, has been intensively investigated. miRNAs are a class of small, 19–22 nucleotides, non-coding endogenous single-stranded RNAs that act as post-transcriptional regulators of specific messenger transcripts (mRNAs), resulting in targeted degradation or suppression of gene expression [25, 26]. More than 4469 miRNAs have been identified in Homo sapiens, of which 1881 are precursors and 2588 are mature (miRBase, Release 21: June 2014) and most of these miRNAs are highly conserved across species. It has been reported that miRNAs are able to control more than 60% of human protein-coding genes [27, 28]. In physiologic conditions miRNAs are key regulators involved in biological processes such as development, proliferation, differentiation, migration, neuroplasticity, survival and death. miRNAs dysregulation contributes to the onset of different pathologies such as heart disease, diabetes, mental disorders and cancer. Because 50% of miRNAs genes are located at genomic sites associated with cancer-specific chromosomal rearrangements and because of the proximity of their genes to chromosomal breakpoints, miRNAs have been associated with tumorigenesis. In some cancer types miRNAs appear to be upregulated and are thus thought to act as oncogenes, while they are downregulated in other types of cancers, which may be indicative of a tumor suppressor function. miRNAs expression is dynamic: many miRNAs are deregulated in early stages of tumor development and upregulated during cancer progression, which underscores the importance of the cellular microenvironment [29]. miRNAs can be used as biomarkers to discriminate cancer from normal tissue, to diagnose the onset of a tumor, to indicate the degree of dissemination and to monitor the response to drug treatments, or as therapeutic targets in the design of a real “miRNA-based therapy” [28]. In silico analyses suggest that *DLL1* is one of the targets of the miRNA-34 family; miRNA-34a maps to the distal region of chromosome 1p which is commonly deregulated or deleted in neuroblastoma (www.mirbase.org). miRNA-34a can antagonize many different oncogenic processes by regulating genes that function in various cellular pathways. The anti-oncogenic activity of miRNA-34a has been demonstrated in cancer cells of the lung [30, 31], pancreas [32, 33], brain [34, 35], ovary [36], prostate [37] as well as in lymphoma and leukemia [38]. miRNA-34a inhibits the propagation properties of tumor-initiating cells derived from medulloblastoma [39] and it is downregulated in glioblastoma tissues, where its overexpression could suppress cell proliferation and induce apoptosis, indicating that this miRNA may act as tumor suppressor also in this type of tumor [40]. miRNA-34b is significantly downregulated in prostate cancer and its reconstitution induced anti-proliferative and antimigratory effects and suppressed tumor growth in an in vivo xenograft nude mouse model, suggesting the tumor suppressor function of this miRNA [41]. Also, in breast cancer, miRNA-34b acts as an oncosuppressor regulating the complex estrogenic pathway, which could lead to the development of new therapeutic strategies [42]. The miRNA-34 family was the most extensively studied miRNAs in neuroblastoma and Welch and colleagues were the first to report that miRNA-34a was generally expressed at lower levels in unfavorable primary neuroblastomas and cell lines compared to normal adrenal tissue. miRNA-34a induced cell cycle arrest, apoptosis, and significantly reduced tumor growth in an in vivo orthotopic murine model of neuroblastoma [35]. Our data indicate that, within the miRNA-34 family, miRNA-34b induced significant downregulation of DLL1 mRNA expression levels, cell differentiation and arrested cell proliferation in IMR-32 neuroblastoma cells. This study identified Notch ligand DLL1 as a new and specific molecular target in childhood neuroblastoma, suggesting that miRNAs could be a novel therapeutic tool to develop an effective strategy to attack “DLL1 positive” neuroblastoma.

## Methods

### Cell lines

The human SH-SY5Y neuroblastoma cell line (DSMZ) was cultured in a 1:1 mixture of Ham’s F12 nutrient and Dulbecco’s modified Eagle’s medium (Sigma-Aldrich) supplemented with 10% fetal bovine serum (FBS, Sigma-Aldrich), 2 mM L-glutamine, 50 mg/mL penicillin, and 100 mg/mL streptomycin (Sigma-Aldrich). The IMR-32 neuroblastoma cell line (DSMZ) and the KELLY neuroblastoma cell line (Sigma-Aldrich) were grown in RPMI medium (Sigma-Aldrich) supplemented with 10% FBS (Sigma-Aldrich), 2 mM L-glutamine, 50 mg/mL penicillin, 100 mg/mL streptomycin (Sigma-Aldrich), and 1× MEM nonessential amino acid solution (Sigma-Aldrich). All the cell lines were grown at 37 °C in a 95% air–5% CO2 humidified incubator.

### siRNAs and miRNAs transient transfection

siRNA probes targeted to the DLL1 ligand were purchased from Dharmacon (Dharmacon, Inc., Lafayette, CO, USA). Human-specific *DLL1* interference was performed using an Accell SMARTpool siRNA mixture containing a mixture of four siRNAs targeting the *DLL1* gene. A non-targeting Accell siRNA pool was used as a control in siRNA transfection experiments. IMR-32 neuroblastoma cells were transfected with Accell siRNAs, using Hi-perfect transfection reagent (Qiagen) in culture medium with 3% normal serum 1 day after seeding. Cells were maintained in culture for three more days after transfection with 20 nM siRNA. IMR-32 neuroblastoma cells were transfected with different miRNA-34 (miRNA-34a, miRNA-34b, miRNA-34c, Qiagen) and miRNA-210 (Qiagen), as internal control, using Hi-perfect transfection reagent (Qiagen) in culture medium with 3% normal serum 1 day after seeding. Cells were maintained in culture for three more days after transfection with 10 nM miRNAs.

### Quantitative real-time PCR

Quantitative real-time PCR (RT-qPCR) was executed as described below. The total RNA was isolated from SH-SY5Y, KELLY and IMR-32 neuroblastoma cells using the RNeasy kit (Qiagen) and digested with the RNase-Free DNase set (Qiagen), according to the manufacturer’s protocol. One microgram of total RNA was transcribed into complementary DNA (cDNA) using murine leukemia virus reverse transcriptase (Promega Italia) and oligo(dT)15-18 as a primer (final volume: 50 μl). The oligonucleotide sequences of the primers used are as follows: N-Myc forward primer 5′-CGA CCA CAAGGC CCT CAG TA-3′, reverse primer 5′-CAG CCTTGG TGT TGG AGG AG-3′; DLL1 forward primer 5′-ACGAATGCTGCTGCTGAAGAGGAGGGA-3, reverse primer 5′-AACTGTCAATAGTGCAACGGCGAC-3′;DLL3 forward primer 5′-AGCGTCACACAATCACGAAG-3′, reverse primer 5′-TGGTATGAACCAGAGCTACCG-3′; DLL4 forward primer 5′-AACTGCCCTTCAATTTCACCT-3′, reverse primer 5′-GCTGGTTTGCTCATCCAATAA-3′; Jagged 1 forward primer 5′-AGACATCGATGAATGCGTCA-3′, reverse primer 5′-CCACAGACGTTGGAGGAAAT-3′; Jagged 2 forward primer 5′-TGGCACTCGCTGTATGAAAG-3′, reverse primer 5′-AGGGCCACATCAATAACCAG-3′; GAPDH forward primer 5′-GAG TCA ACG GAT TTG GTC GT-3′, reverse primer 5′-TTG ATT TTG GAG GGA TCT CG-3′. Amplification and detection were performed with the iCYCLER iQ Real Time PCR Detection System (BioRad Italia, Milan, Italy); the fluorescence signal was generated by SYBR Green I. Samples were run in triplicate in a 25 μl reaction mix containing 12.5 μl 2× SYBR Green Master Mix (BioRad Italy), 12.5 pmol of each forward and reverse primer and 2 μl of diluted cDNA. The PCR program was initiated by 10 min at 95 °C followed by 40 cycles, each for 15 s at 95 °C and 1 min at 60 °C. Gene expression levels were normalized to GAPDH expression and data are presented as the fold change in target gene expression in drug-treated cells normalized to the internal control gene (GAPDH) and relative to untreated cells. Results were estimated as Ct values; the Ct was calculated as the mean of the Ct for the target gene minus the mean of the Ct for the internal control gene. The Ct represented the mean difference between the Ct of untreated cells minus the Ct of treated cells. The *N*-fold differential expression in the target gene of drug-treated cells compared with untreated cells was expressed as 2^- ∆∆Ct^. Data analysis and graphics were performed using Graph Pad Prism 5 software and the results of experiments were run in triplicate.

### Western blot analysis

Total cell lysates were prepared by scraping the cells in lysis buffer (50 mM Tris pH 7.6, 150 mM NaCl, 2 mM EDTA, 0.5% NP40 with a cocktail of protease inhibitors). For Western blot analysis, 15 μg of total proteins were electrophoresed onto 10% SDS-PAGE and transferred to nitrocellulose paper. Filters were incubated with anti-Notch 1 antibody (Sigma-Aldrich; 1:1000), anti-Neuronal Nuclei antibody (Millipore; 1:1000), anti-β III tubulin (Promega; 1:1000) and anti-Glyceraldehyde-3-phosphate dehydrogenase antibody (Millipore; 1:500) as loading control. After washing, membranes were incubated with HRP-conjugated antimouse and antirabbit secondary antibody (Dako; 1:1500) and a chemiluminescence blotting substrate kit (Amersham Biosciences) was used for immunodetection. Evaluation of immunoreactivity was performed on immunoblots by densitometric analysis using the Quantity One analysis software (BioRad Laboratories GmbH).

### Immunofluorescence and morphometric analysis

Immunofluorescence and morphometric analyses were executed as described below. SH-SY5Y cells were plated with a density of 75 × 10^3^/well in a 24 wells plate, grown on a glass coverslip (coated with poly-l-lysine, Sigma-Aldrich); IMR-32 cells were plated with a density of 50 × 10^3^/well and grown on a glass coverslip coated with collagen IV (BD Bioscience). Cells were fixed in ice-cold methanol (Sigma-Aldrich), then washed and incubated in Phosphate Buffered Saline (PBS, Sigma-Aldrich) containing 1% of Bovine Serum Albumin (BSA, Sigma-Aldrich) and 0.2% Triton X 100 overnight at 4 °C with a polyclonal anti-β III tubulin (Sigma-Aldrich, 1:600), monoclonal anti-Notch 1 (Sigma-Aldrich, 1:1000). After rinses, cells were incubated with Alexa Fluor® 488 (Life Technologies, 1:400) anti rabbit secondary antibody and CY™3-conjugated anti-mouse secondary antibody (Jackson Immunoresearch Laboratories INC.,1:500) in PBS for 1 h at room temperature. For morphological evaluation, slices were mounted and examined by a ZEISS LSM 510 META confocal laser-scanning microscope (Carl Zeiss, Germany). Images were processed using LSM5 image examiner software (Zeiss). The percentage of morphologically differentiated cells was determined by analyzing at least 10 fields for each treatment; cells with neurites ≥50 μm in length were considered as differentiated.

### Flow Cytometry for analysis of cell cycle and apoptosis

For cell cycle analysis, cells were harvested at the completion of the miRNAs treatments and washed with phosphate-buffered saline (PBS; pH 7.4) before being fixed with 70% ethanol on the wheel for 15 min at 4 °C. Subsequently, the cells were centrifuged at 4500 rpm for 5 min at 4 °C, washed with phosphate-buffered saline and were resuspended in 600 μl of 0,1% sodium citrate (Sigma-Aldrich), 50 μg/ml of propidium iodide (PI, Sigma-Aldrich) and 10 μg/ml of Ribonuclease A (Sigma-Aldrich) for staining cellular DNA. The cellular DNA content was then analyzed using a MACS Quant Flow Cytometer (Miltenyi Biotec). Data analysis was carried out using FlowJo software. For the apoptosis analysis, after treatment with miRNAs 10 nM, cells were washed with PBS and stained with Annexin V-FITC and PI using the Apoptosis Detection Kit (Bender Medsystems) according to the manufacturer’s protocol. Annexin-positive cells were counted using a MACS Quant Flow Cytometer (Miltenyi Biotec) within 1 h after staining. Data analysis was carried out using FlowJo software.

### Statistical analysis

Statistical analyses were performed by one-way analysis of variance followed by Bonferroni’s multiple comparison test as post hoc analysis. Data are presented as the mean ± Standard Error Mean (S.E.M). Probabilities <0.05 and <0.001 were considered a significant difference.

## Results

### DLL1 is the notch pathway component highly expressed in *MYCN* amplified neuroblastoma cells

**Fig. 1:**
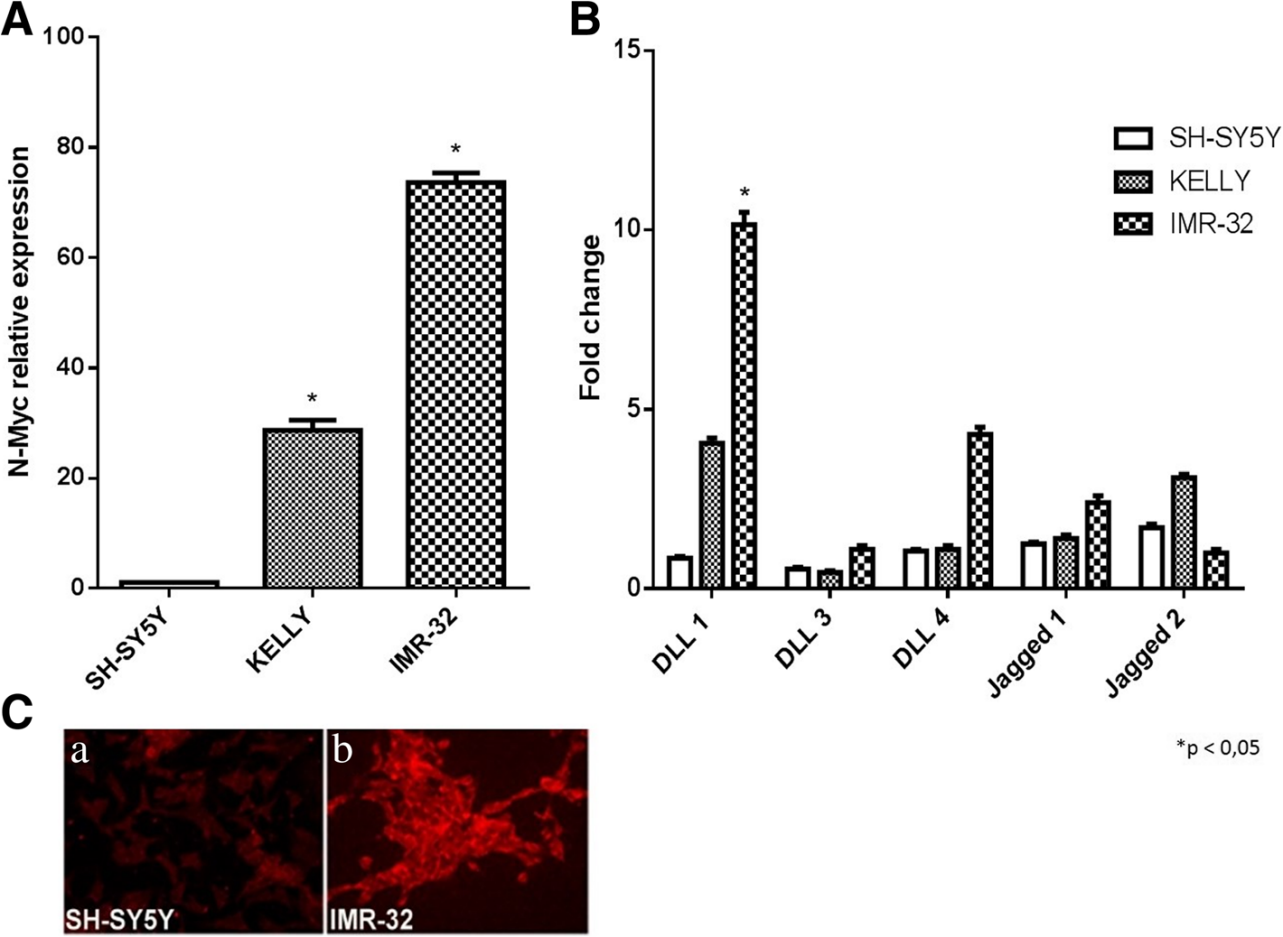
Analysis of Notch ligands expression in neuroblastoma cells with different *MYCN* gene amplification. **a** RT-qPCR analysis of N-Myc mRNA expression levels in SH-SY5Y, KELLY and IMR-32 neuroblastoma cell lines. **b** RT-qPCR analysis of the five mammalian Notch ligands in SH-SY5Y, KELLY and IMR-32 neuroblastoma cell lines. **c** Immunofluorescence analysis of DLL1 protein expression in SH-SY5Y and IMR-32 neuroblastoma cells. *p < 0.05 vs SH-SY5Y cell line

At first, the role of Notch pathway components was investigated in neuroblastoma cell lines with different *MYCN* gene amplification degree, which is related to aggressiveness and malignant phenotype. RT-qPCR was performed in three cell lines: SH-SY5Y cell line with low degree of *MYCN* amplification, IMR-32 and KELLY with high degree of *MYCN* amplification [43].The N-Myc mRNA expression levels were established by RT-qPCR, that confirmed a barely detectable N-Myc expression in SH-SY5Y cells and a higher N-Myc expression levels in KELLY and IMR-32 cells (Fig. 1a). The mRNA expression levels of three Notch receptors (Notch 1, Notch 2 and Notch 3) and five Notch ligands (Delta-like 1, Delta-like 3, Delta-like 4, Jagged 1 and Jagged 2) were analyzed by RT-qPCR and we identified DLL1 as the Notch pathway component highly expressed in IMR-32 cells (Fig. 1b). Receptors were also analyzed but uniformity was not found among the three cell lines (Additional file 1). DLL1 protein expression was also analyzed by immunofluorescence that confirmed the high ligand levels in IMR-32 cells compared with SH-SY5Y cell line (Fig. 1c).

### High DLL1 ligand expression levels are associated with notch pathway activation in IMR-32 neuroblastoma cells

**Fig. 2:**
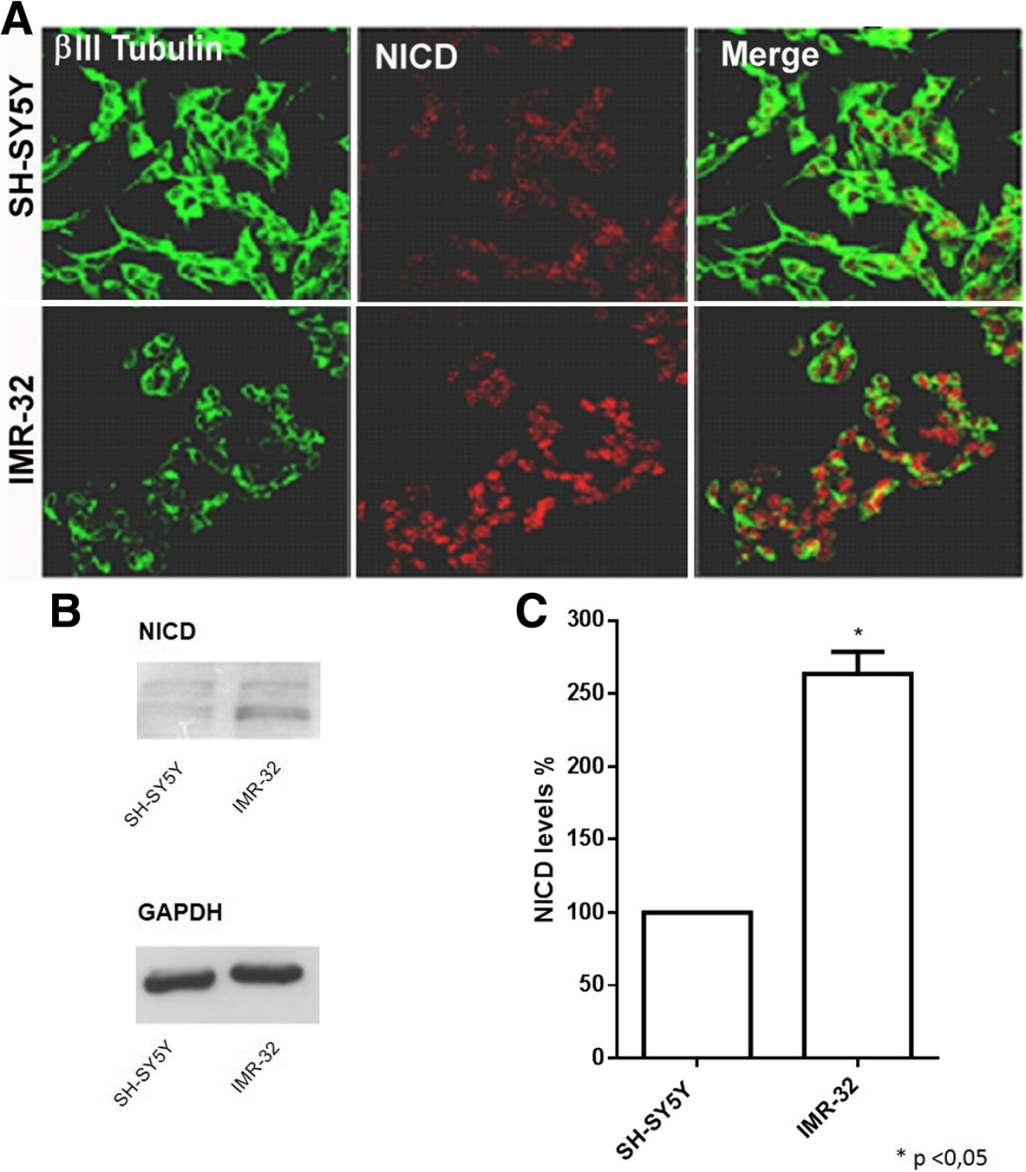
Analysis of Notch pathway activation in neuroblastoma cells with different *MYCN* gene amplification. SH-SY5Y and IMR-32 cells were chosen as the two cell lines with the lowest and the highest N-Myc mRNA expression. **a** Confocal analysis of NICD expression levels in SH-SY5Y and IMR-32 cells. β III tubulin antibody (*green*) was used as neuron marker; NICD antibody (*red*) showed a typical nuclear localization, indicative of Notch activation. **b** Western blot analysis of NICD protein expression in SH-SY5Y and IMR-32 cells. **c** Densitometric analysis of NICD expression levels in SH-SY5Y and IMR-32 cells using Quantity One analysis software**.** *p < 0.05 vs SH-SY5Y cell line

To verify if DLL1 increased levels leads to an effective Notch pathway activation, the expression of NICD protein (Notch Intracellular Domain, Notch 1 activated form) in SH-SY5Y and IMR-32 neuroblastoma cells was analyzed. SH-SY5Y and IMR-32 cells were cultured for 48 h in complete medium and then analyzed by immunofluorescence. A stronger Notch activation was found in IMR-32 cells compared to SH-SY5Y (Fig. 2a). This result was confirmed by Western blot and densitometric analysis, showing a significant increase of NICD protein level in IMR-32 cell line (Fig. 2b, c).

### Selective DLL1 downregulation by siRNA induces neuroblastoma cell differentiation

**Fig. 3:**
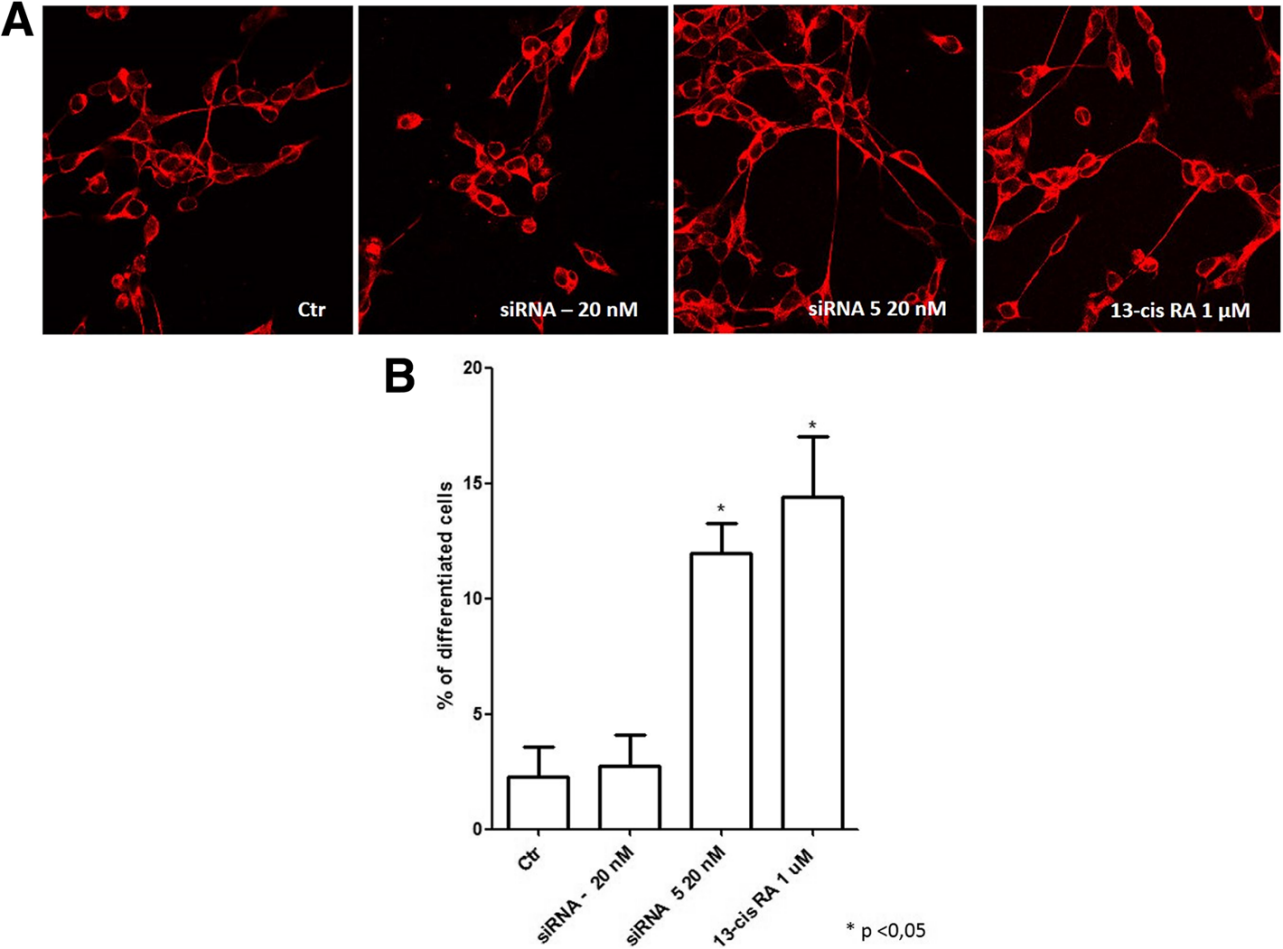
Analysis of siRNA DLL1 and 13-cis retinoic acid treated IMR-32 neuroblastoma cell line. **a** Confocal analysis of siRNA DLL1 and 13-cis retinoic acid in IMR-32 neuroblastoma cells after 3 days of transfection. β III tubulin antibody was used as neuron marker. **b** Morphometric analysis of cell differentiation induced by siRNA DLL1 and 13-cis retinoic acid. Neuronal differentiation was evaluated by measuring neurite length. The percentage of differentiated cells was calculated, considered as cells with neurites ≥50 μM in length in IMR-32 cells. *p < 0.05 vs control

Since DLL1 was identified as the Notch pathway component most expressed in IMR-32 neuroblastoma cells, and since DLL1 increased levels lead to an effective Notch pathway activation, we investigated *DLL1* molecular targeting by observing the effects of *DLL1* gene silencing via RNA interference technique in neuroblastoma cells. First, the efficacy of various siRNAs at different concentrations (5 nM, 10 nM, 20 nM and 40 nM) was assessed in terms of their ability to induce significant DLL1 downregulation. We identified siRNA 5 at the concentration of 20 nM as the most capable to significantly downregulate DLL1 mRNA expression levels; siRNA – (non-targeting siRNA pool) 20 nM was used as scrambled negative control (Additional file 2). To study the effect of siRNA on neuroblastoma cell differentiation, IMR-32 cells were transfected with siRNA 5 20 nM and cells were analyzed by immunofluorescence. Confocal microscopy analysis showed that DLL1 downregulation by siRNA 5 induced neuronal differentiation, characterized by changes in cell morphology such as longer neurites, as obtained with 13-cis retinoic acid (Fig. 3a). Retinoic acid was used as a positive control because it is a potent cellular differentiation agent able to induce growth inhibition and cell differentiation in normal and cancer cell types. Retinoic acid is currently used in neuroblastoma treatment, in association with standard therapeutic protocols, as neoadjuvant agent [44]. It is interesting and significant that DLL1 downregulation induced a cell differentiation comparable to the effect obtained with 13-cis retinoic acid. A quantitative analysis of morphological differentiation confirmed that DLL1 downregulation induced a statistically significant increase in the number of differentiated cells (Fig. 3b).

### DLL1 is downregulated in IMR-32 neuroblastoma cells treated with miRNA-34b

**Fig. 4:**
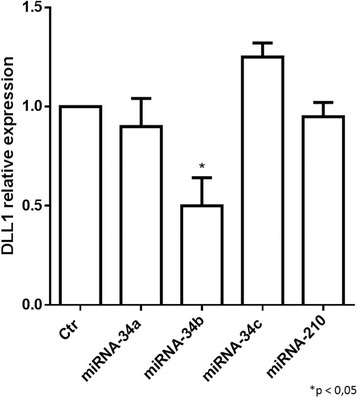
RT-qPCR analysis of DLL1 mRNA expression levels in IMR-32 neuroblastoma cell line. Analysis of DLL1 mRNA expression levels after Authors’ contribution3 days of transfection with all miRNA-34 family members and with miRNA-210. *p < 0.05 vs control

In silico analysis using Target Scan 6.2 (Released June 2012) predict that the *DLL1* is a potential target of miRNA-34 family members. IMR-32 neuroblastoma cells were transfected with miRNA-34 family members (miRNA-34a, miRNA-34b, miRNA-34c) and miRNA-210 as internal control. miRNA-210 was chosen as internal control because in silico analysis has shown that it is not correlated to Notch signaling. miRNA-210 expression is related to diseases such as lung adenocarcinoma [45] and cerebral ischemia [46]. Cells were transfected with the miRNAs at three different concentrations (5 , 10 and 20 nM) and RT-qPCR analysis of DLL1 mRNA expression levels showed that in cells treated with miRNA-34b, at the concentration of 10 nM, it was observed a significant downregulation of DLL1 mRNA expression levels (Fig. 4). This data suggest an important role of miRNA-34b in DLL1 downregulation. At concentrations of 5 nM and 20 nM, no significant differences between control and treated cells were found. We observed that the 5 nM concentration had no effects, while 20 nM concentration was toxic for cells which showed clear signs of cell suffering, and the analysis on cell extracts of RNA revealed no differences in expression levels.

### miRNA-34b induces differentiation in IMR-32 neuroblastoma cells

**Fig. 5:**
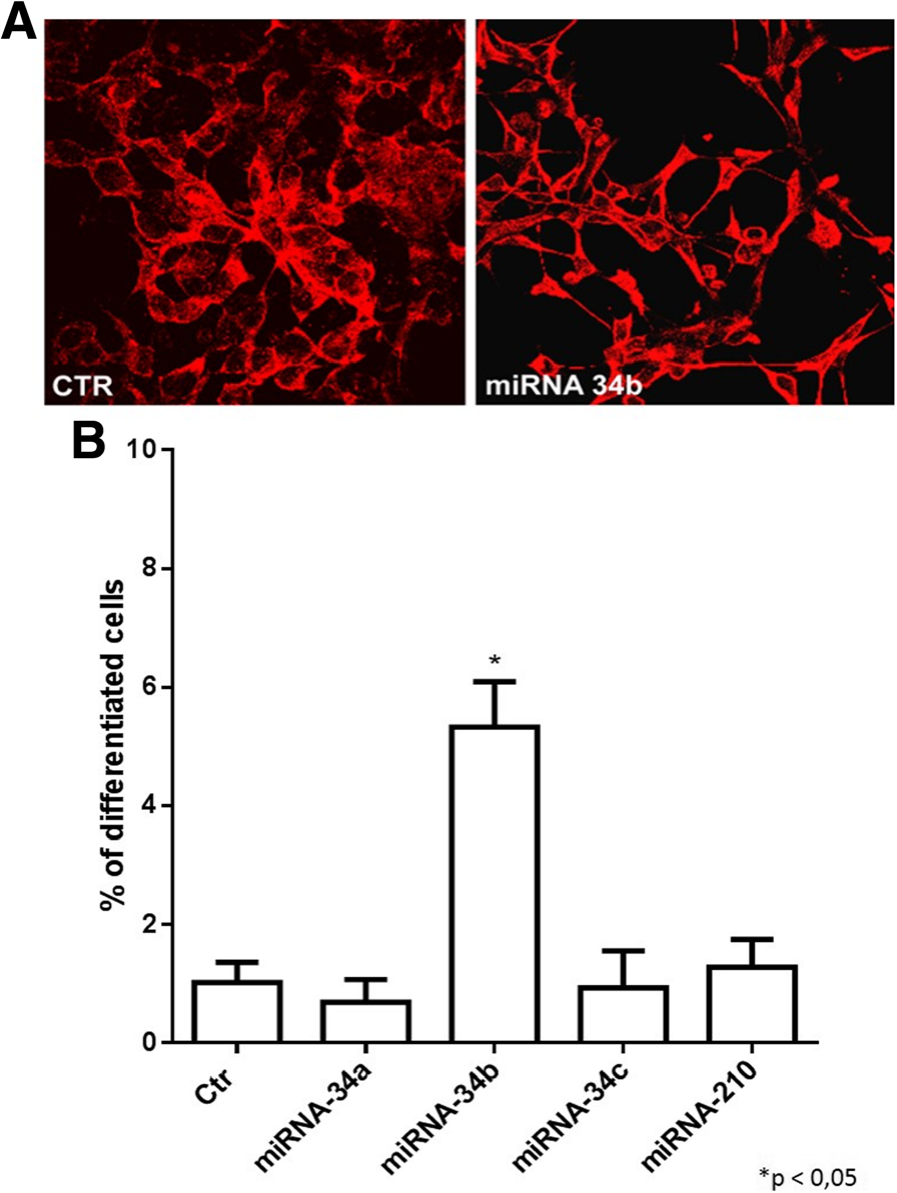
Analysis of miRNA-34b transfection in IMR-32 neuroblastoma cells line. **a** Confocal analysis of miRNA-34b transfection in IMR-32 neuroblastoma cells after 3 days of transfection. β III tubulin antibody was used as neuron marker. **b** Morphometric analysis of cell differentiation induced by miRNAs transfection. IMR-32 neuroblastoma cells underwent 3 days of transfection and then neuronal differentiation was evaluated by measuring neurite length. The percentage of differentiated cells was calculated, considered as cells with neurites ≥50 μM in length in IMR-32 cells. *p < 0.05 vs control

**Fig. 6:**
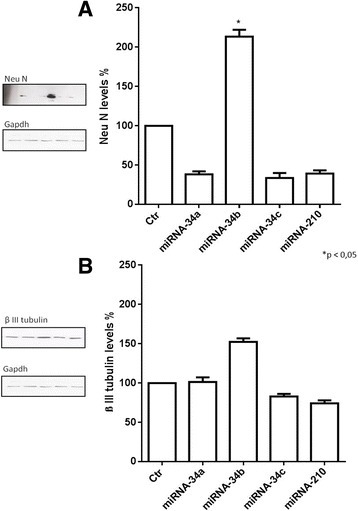
Western Blot analysis of IMR-32 neuroblastoma cell line. Western Blot analysis of Neu N (**a**) and β III tubulin (**b**) protein expression levels after 3 days of miRNAs transfection. *p < 0.05 vs control

The possible morphological changes after miRNAs transfection on IMR-32 neuroblastoma cells were evaluated. Analysis by confocal microscopy showed that miRNA-34b transfection induced significant cellular differentiation, characterized by longer neurites (Fig. 5a), increasing the number of differentiated cells compared to control (Fig. 5b). Morphological analysis was accompanied by biochemical patterns resembling the neuronal phenotype. We therefore measured the levels of neuronal specific markers including Neuronal Nuclei protein (Neu N) and β III tubulin by Western blot. Protein expression levels of Neu N were strongly increased after miRNA-34b treatment (Fig. 6a); the protein expression levels of β III tubulin were also increased even if the data was not statistically significant (Fig. 6b). These results suggest that miRNA-34b may function as a cell fate regulator, which modulates early cell differentiation in IMR-32 neuroblastoma cells.

### miRNA-34 family members induce cell proliferation arrest in IMR-32 neuroblastoma cells

**Fig. 7:**
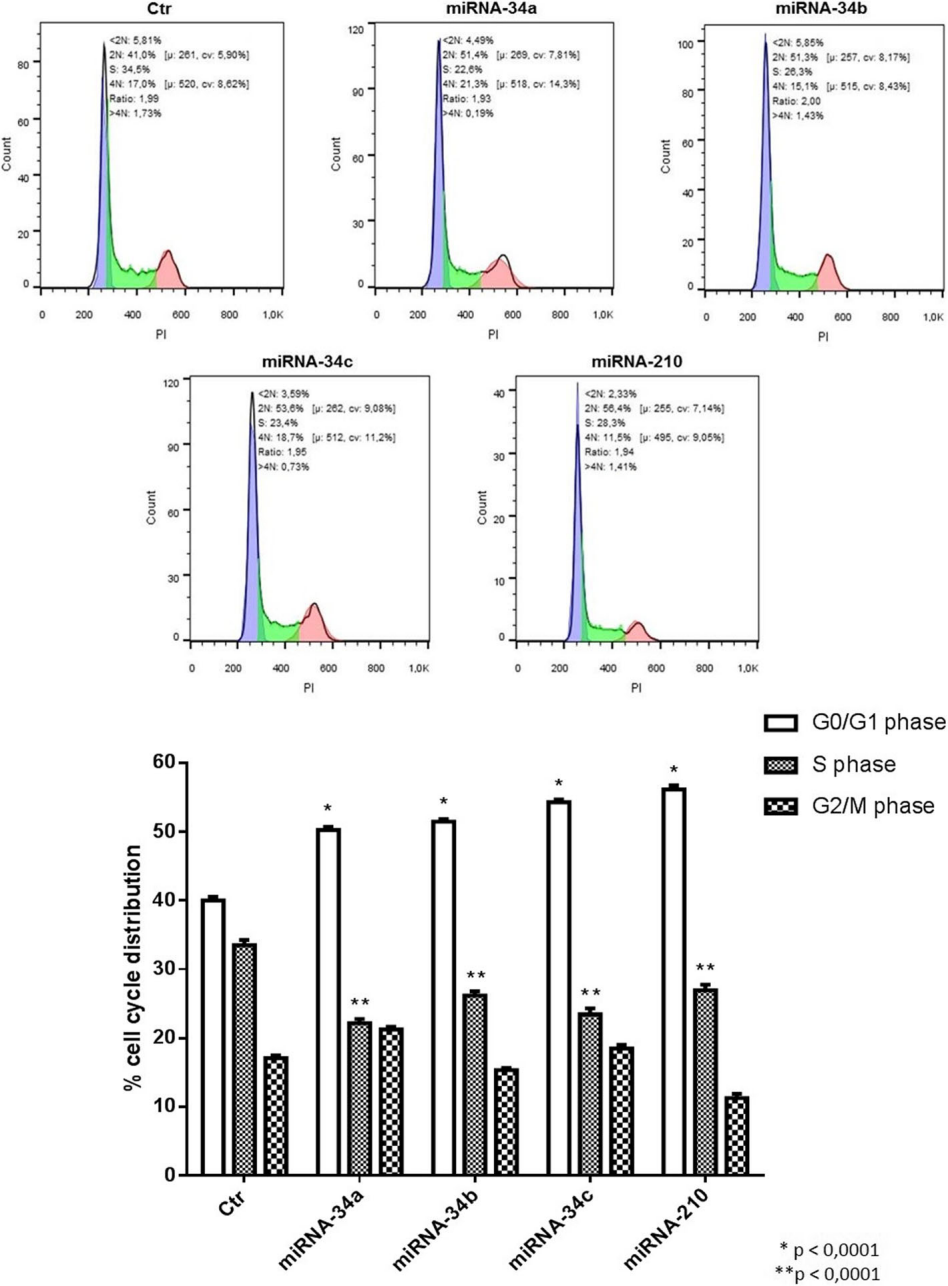
Cell cycle analysis of IMR-32 neuroblastoma cell line. Cell cycle analysis, by Flow Cytometer, of IMR-32 cells after 3 days of miRNAs transfection. *p < 0.0001 vs G0/G1 phase control. **p < 0.0001 vs S phase control

The ability of miRNAs to arrest cell proliferation was assessed by cell cycle analysis. miRNA-34b induced an increase in the number of cells in G0/G1 phase (*p < 0.001) and a decrease in the number of cells in S phase (**p < 0.001) of the cell cycle, compared to respective control. Other members of miRNA-34 family have such effects. The data indicate that the treatment with all miRNA-34 family members blocked the cells in the G0/G1 phase, decreasing the number of proliferating tumoral cells (Fig. 7). The effect of miRNA-34b is peculiar and specific for differentiation pathway and not for cell proliferation arrest.

### miRNA-34 family members do not induce apoptosis in IMR-32 neuroblastoma cells

**Fig. 8:**
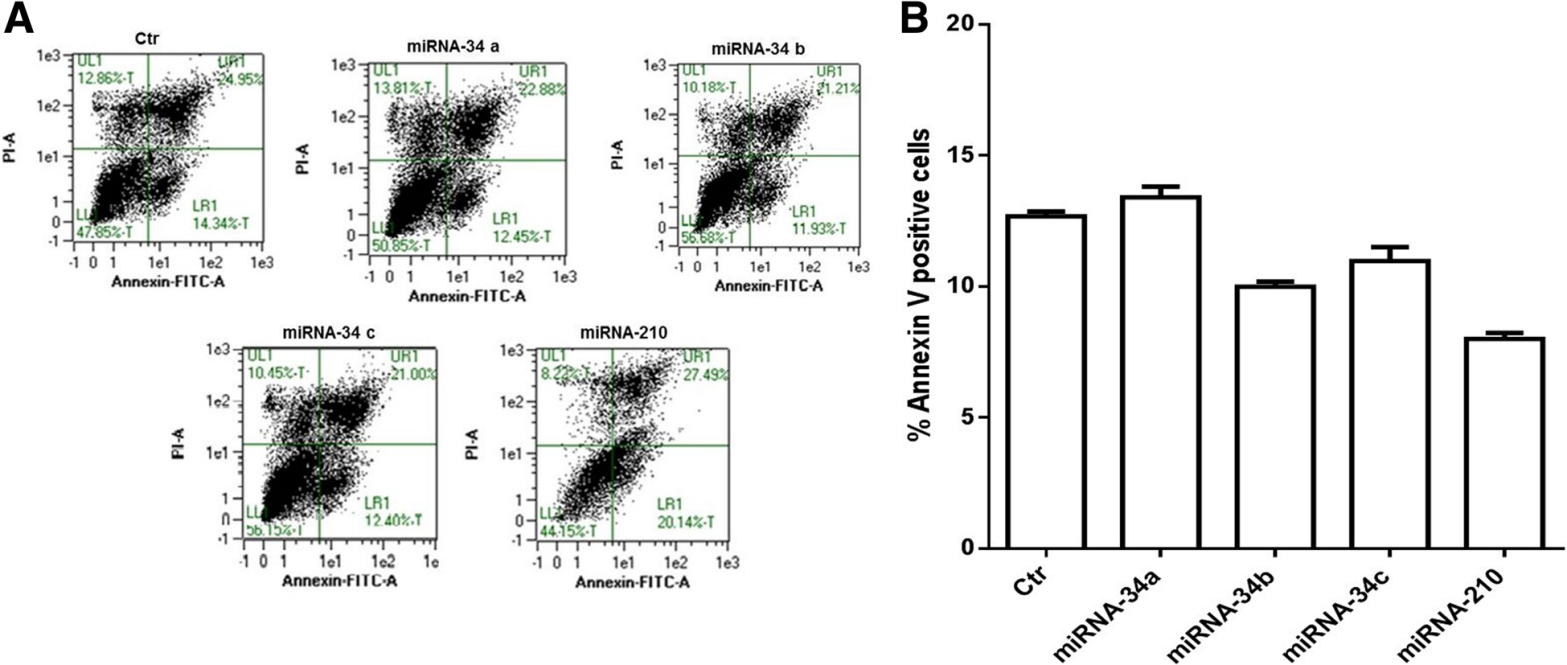
Apoptosis analysis of IMR-32 neuroblastoma cell line. **a** Annexin V/PI double staining and flow cytometry assays. *X-axis* indicates the number of Annexin V-FITC-stained cells. *Y-axis* indicates the number of PI-stained cells. **b** Determination of apoptosis percentages based on the accumulation of Annexin V-positive cells

Finally a possible apoptotic effect of miRNAs on IMR-32 neuroblastoma cells was analyzed. Cytofluorimetric analysis, by Annexin V-positive cells, revealed that members of the miRNA-34 family do not induce apoptosis in the IMR-32 neuroblastoma cell line (Fig. 8a, b). These data suggest that miRNAs treatments drive the cells in G0/G1 phase towards a differentiation profile instead to cell death.

## Discussion

In the present study Notch ligand DLL1 emerged as a novel molecular target in childhood neuroblastoma and miRNAs could be an innovative tool to attack “DLL1 positive” tumors. The DLL1 ligand was observed to be the most expressed Notch pathway component in *MYCN* amplified neuroblastoma cells and its downregulation, with miRNAs, induced differentiation and an arrest of cell proliferation. Recently, the focus of cancer research has been the identification of new molecular targets which will permit to act with more specific and effective drugs, thereby paving the path towards innovative therapeutic strategies. The involvement of the Notch pathway in tumors, including neuroblastoma, has previously been described; our group has demonstrated that Notch activation is responsible for growth and proliferation of neuroblastoma cells, while its inhibition causes proliferation arrest and cell differentiation [8]. DLL1 is a transmembrane ligand of the Notch signaling pathway that is also overexpressed in other types of tumor, such as choriocarcinoma [20] and medulloblastoma [39], indicating a probable strong involvement of this Notch pathway component in the onset of such tumors. Furthermore, DLL1 is not only implicated in tumorigenesis, it is also involved in sympathetic ganglia development where the protein maintains the proliferation state of embryonal cells [47, 48]. In the present study DLL1 was found to be highly expressed in IMR-32 cells, a cell line with a high degree of *MYCN* gene amplification. The *MYCN* proto-oncogene plays an important role in neuroblastoma development; about 30%-40% of advanced stage tumors exhibit *MYCN* amplification, which has been correlated to rapid tumor progression, drug resistance and poor outcome [49, 50]. Thus, these data probably suggest that *DLL1* expression is dependent on the degree of *MYCN* gene amplification, although further work is needed to demonstrate this. Zhao and colleagues had previously demonstrated that Delta-like 3 (*DLL3*) is a direct transcriptional target of N-Myc, identifying a new “N-Myc-DLL3” pathway as a mediator of neural development in brain [51]. Recently, miRNAs have emerged as a new promising therapy in many different diseases, including cancers, thanks to their ability to modulate selective molecules at post-transcriptional level [52, 53]. miRNAs act as fine regulators of cancer development, functioning as oncogenes or tumor suppressor, and they can be used to form the new “miRNA-based therapy” for cancer [54, 55]. In silico analysis showed that DLL1 ligand is one of the targets of miRNA-34 family, which is known for its potential tumor suppressing role in several cancers including neuroblastoma, glioblastoma, and medulloblastoma. In neuroblastoma tissues and cell lines, members of the miRNA-34 family are expressed at very low levels and their ectopic expression induce cell cycle arrest, apoptosis, and a reduction of tumor growth in vivo [56]. The data suggest a role for miRNA-34a as a potential tumor suppressor in neuroblastoma [35], and for miRNA-34b as an oncosuppressor in in vitro prostate cancer models and breast cancer [41, 42]. In this study miRNA-34b was able to significantly downregulate DLL1 ligand and to induce neuronal differentiation, much more than other components of the miRNA-34 family. This divergence may be due to the fact that the sequences of miRNA-34a and miRNA-34b differ for few nucleotides and the different actions of miRNAs probably depend on the tumor microenvironment, cell lines used, as well as the choice of in vivo tumoral model. One of the mechanisms of action of miRNAs is the induction of apoptosis, through the activation of a caspase-dependent apoptotic pathway, and the inhibition of proliferation [36]. The results obtained in IMR-32 neuroblastoma cell line demonstrate that members of the miRNA-34 family are able to induce a significant arrest of tumor cell proliferation but not to induce apoptosis. Probably cells blocked in G0/G1 phase move towards a path of cell differentiation and not apoptotic death. Whether miRNAs induce apoptosis presumably depends on the cellular context and on the expression levels of the miRNA-34 target proteins involved in the regulation of apoptosis.

## Conclusions

This study was conducted in order to identify the Notch pathway components highly involved in neuroblastoma genesis and progression. DLL1 emerged to be the ligand highly expressed in cell lines with high *MYCN* gene amplification, indicative of increased degree of malignancy. Downregulation of the ligand inhibited cell proliferation and induced differentiation, suggesting a pro-oncogenic role for *DLL1*. The ligand DLL1 was also shown to be a potential target of miRNA-34b, opening the possibility to use miRNAs-based therapy. This idea could be supported by the observation that miRNAs treated cells also showed proliferation arrest and neurite elongation. In conclusion, the data collected point to DLL1 Notch ligand as a new and attractive molecular target in childhood neuroblastoma, and we propose miRNAs as new potential therapy to act on high DLL1 expression. There is strong evidence in the literature supporting the idea that miRNAs could be a new, potentially more effective and specific therapeutic tools, compared with current medical strategies. This study also contributes to the knowledge concerning the role of the Notch pathway in neuroblastoma, shedding light on mechanisms and molecules involved in neuroblastoma pathogenesis.

## Additional files

Additional file 1: Analysis of Notch receptors expression in neuroblastoma cells with different *MYCN* gene amplification. RT-qPCR analysis of the three mammalian Notch receptors (Notch 1, Notch 2, and Notch 3) in SH-SY5Y, KELLY and IMR-32 neuroblastoma cell lines. *p < 0, 05 vs SH-SY5Y cell line. (JPEG 216 kb)

Additional file 2: Analysis of the efficacy of various siRNAs at different concentrations on DLL1 mRNA expression levels in IMR-32 neuroblastoma cells. RT-qPCR analysis of siRNA – (non-targeting siRNA pool), siRNA 2 and siRNA 5 at four different concentrations (5 nM, 10 nM, 20 nM, 40 nM) in IMR-32 neuroblastoma cell line. *p < 0, 05 vs control. (JPEG 197 kb)

## Abbreviations

DLL1: Delta-like 1
DLL3: Delta-like 3
DLL4: Delta-like 4
miRNAs: microRNAs
