# Supplementary figures and images for: Notch ligand Delta-like 1 as a novel molecular target in childhood neuroblastoma
Source: BMC Cancer. 2017 May 19;17:352. doi: 10.1186/s12885-017-3340-3 (PMC5438559; doi:10.1186/s12885-017-3340-3)

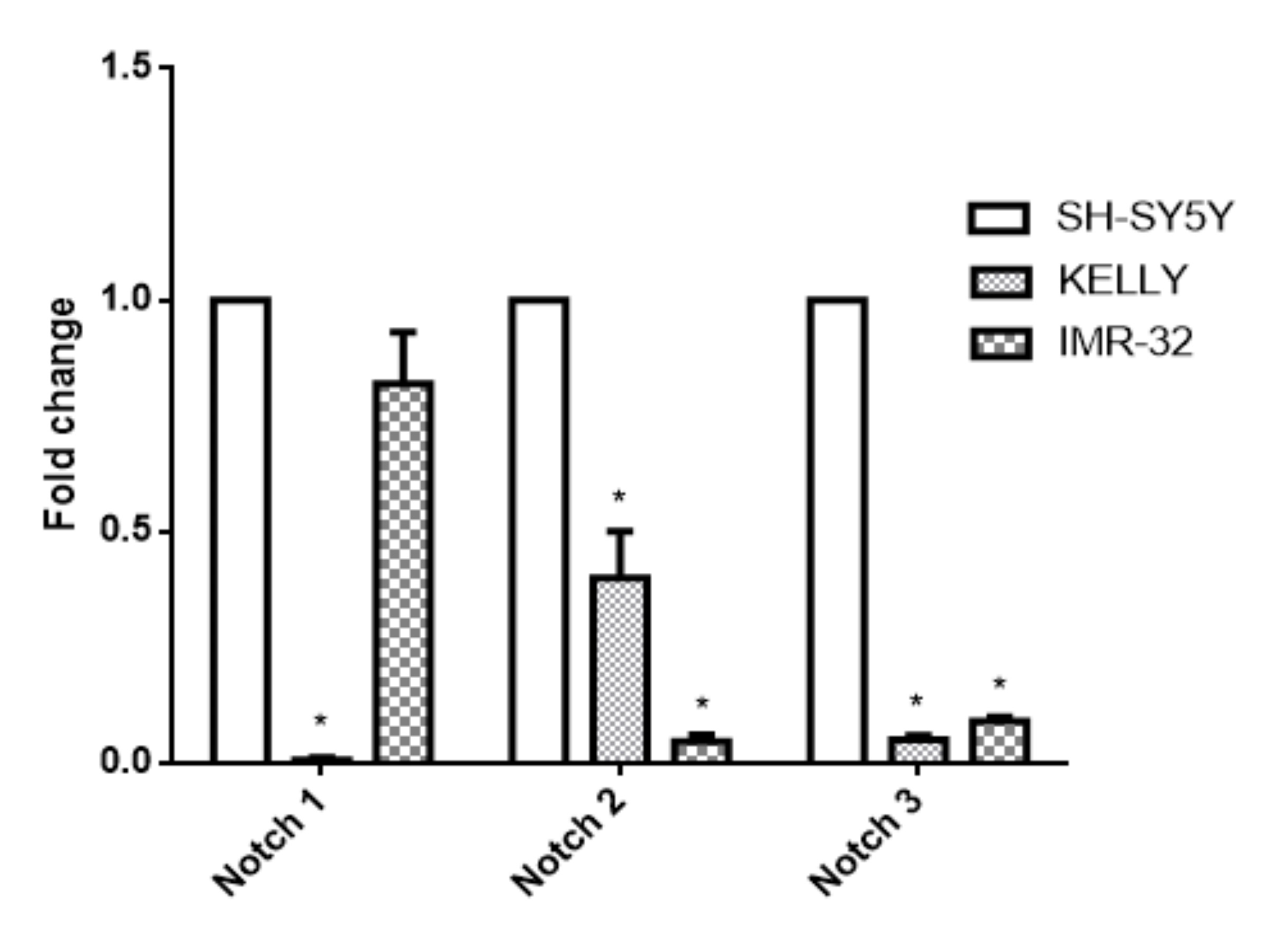

Supplement: Supplementary file 1 — Analysis of Notch receptors expression in neuroblastoma cells with different MYCN gene amplification. RT-qPCR analysis of the three mammalian Notch receptors (Notch 1, Notch 2, and Notch 3) in SH-SY5Y, KELLY and IMR-32 neuroblastoma cell lines. *p < 0, 05 vs SH-SY5Y cell line. (JPEG 216 kb) [file 12885_2017_3340_MOESM1_ESM.jpg]

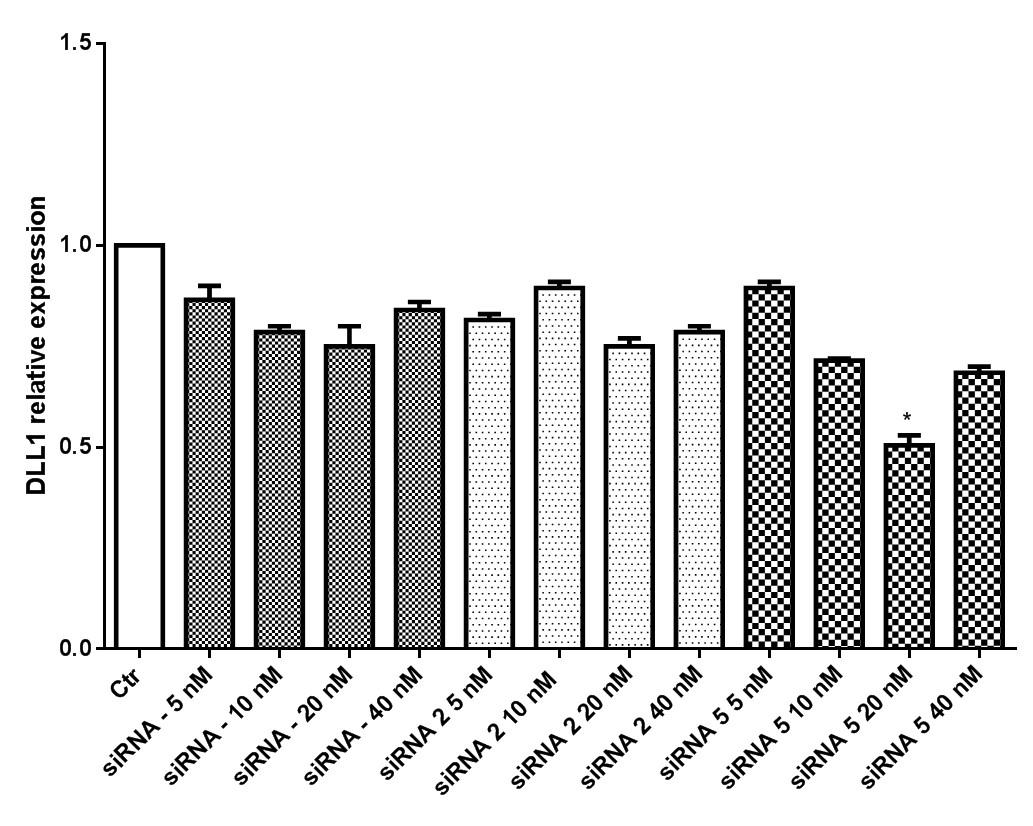

Supplement: Supplementary file 2 — Analysis of the efficacy of various siRNAs at different concentrations on DLL1 mRNA expression levels in IMR-32 neuroblastoma cells. RT-qPCR analysis of siRNA – (non-targeting siRNA pool), siRNA 2 and siRNA 5 at four different concentrations (5 nM, 10 nM, 20 nM, 40 nM) in IMR-32 neuroblastoma cell line. *p < 0, 05 vs control. (JPEG 197 kb) [file 12885_2017_3340_MOESM2_ESM.jpg]
